# Fermented Rapeseed Meal as a Dietary Intervention to Improve Mineral Utilization and Bone Health in Weaned Piglets

**DOI:** 10.3390/ani14182727

**Published:** 2024-09-20

**Authors:** Anna Czech, Katarzyna Woś, Karol Pachciński, Siemowit Muszyński, Michał Świetlicki, Ewa Tomaszewska

**Affiliations:** 1 Department of Biochemistry and Toxicology, Faculty of Animal Sciences and Bioeconomy, University of Life Sciences in Lublin, 20-950 Lublin, Poland; kpachcinski@gmail.com; 2 Department of Biophysics, Faculty of Environmental Biology, University of Life Sciences in Lublin, 20-950 Lublin, Poland; siemowit.muszynski@up.lublin.pl; 3 Department of Applied Physics, Faculty of Mechanical Engineering, Lublin University of Technology, 20-618 Lublin, Poland; m.swietlicki@pollub.pl; 4 Department of Animal Physiology, Faculty of Veterinary Medicine, University of Life Sciences in Lublin, 20-950 Lublin, Poland; ewarst@interia.pl

**Keywords:** fermented rapeseed meal, dietary supplement, bones, piglets

## Abstract

**Simple Summary:**

This study explored how adding fermented rapeseed meal to the diet of young piglets affects their ability to absorb essential minerals and the strength of their bones. It is known that microorganisms used in the fermentation process synthesize enzymes that enhance the bioavailability of macro- and microminerals essential for proper bone formation and growth. We introduced different levels of fermented rapeseed meal into piglets’ diets before weaning and continued feeding it to them until they were 42 days old. We found that moderate amounts of fermented rapeseed meal helped the piglets absorb key minerals like calcium, phosphorus, and magnesium more effectively. This, in turn, led to stronger bones with a better mineral content and stiffness. Our findings suggest that fermented rapeseed meal could be a valuable addition to piglets’ diets, improving their growth and bone health. This research may help raise healthier animals and potentially reduce the need for synthetic mineral supplements, which can be both costly and environmentally harmful.

**Abstract:**

This study examined the effects of incorporating fermented rapeseed meal (FRSM) into the diet of newly weaned piglets on mineral digestibility and bone health. Experimental diets containing varying levels of FRSM (8%, 12%, 15%, and 25%) were introduced to the piglets at 18 days of age, prior to weaning at 28 days. These diets were continued until the piglets were euthanized at 42 days of age. Mineral absorption was assessed using the apparent total tract digestibility (ATTD) method and blood plasma element analysis, while bone mineral content and mechanical properties were evaluated through densitometry and three-point bending tests. The results showed that intermediate levels of FRSM (12–15%) significantly enhanced the digestibility of key minerals, including phosphorus, calcium, magnesium, copper, zinc, and iron. This improvement was linked to increased femoral mineral content and bone stiffness, as well as a higher yield point, likely due to enhanced collagen synthesis. Additionally, there was an increase in bone fracture load and fracture stress, potentially due to changes in the organization of the bone mineral phase, as no changes in bone mid-shaft mineral density or geometry were observed. These findings suggest FRSM as a promising dietary component for improving mineral bioavailability and bone health in piglets.

## 1. Introduction

The immature digestive tract of growing pigs necessitates that piglets receive feed in which nutrients and minerals are highly digestible, along with additives that support intestinal health [1]. Weaning is a critical period for piglets, during which a reduction in *Lactobacillus* bacteria and a loss of microbial diversity in the digestive tract are commonly observed, increasing their susceptibility to pathogenic bacteria such as enterotoxigenic strains of *E. coli*, a leading cause of gastrointestinal diseases [2]. Induced inflammatory processes in the intestinal epithelium can hinder the absorption of nutrients and minerals, leading to deficiencies that disrupt overall metabolism, including proper bone development [3].

The skeletal system is crucial to various physiological functions: it supports body weight, enables movement, protects the internal organs, and serves as a critical attachment point for muscles [4,5]. Calcium and phosphorus are essential for proper bone mineralization, and their deficiency can result in inadequate mineralization in growing animals. This not only reduces bone mass but also increases the risk of bone and fractures, reducing growth performance. The development, formation, and stability of bones heavily rely on the homeostasis of calcium and phosphorus, which is regulated by factors such as intestinal absorption and bone storage [6,7]. Other minerals, including magnesium, zinc, manganese, copper, and iron, are also vital for bone health. Magnesium contributes to bone structure and the regulation of calcium metabolism; zinc is essential for bone growth and development, with zinc deficiencies potentially leading to growth retardation and skeletal abnormalities; copper is crucial for the cross-linking of collagen and elastin, which are important for bone strength and elasticity; and iron is necessary for the proliferation and differentiation of osteoblasts and osteoclasts [8].

The source of minerals in animal feed is crucial for regulating mineral metabolism in the body. Traditionally, feed mixtures were supplemented with inorganic mineral sources; however, the absorption of these inorganic forms has proven to be inefficient [9]. Today, there are various methods for enhancing mineral absorption, including the use of feed enzymes like phytase, non-starch polysaccharide (NSP)-hydrolyzing enzymes, probiotics, prebiotics, and acidifiers [10].

One emerging solution in livestock nutrition is the use of fermented feed components [11,12,13]. Fermented rapeseed meal (FRSM) has garnered particular attention as a valuable protein and energy source for livestock. The lactic acid bacteria involved in the fermentation process confer pro- and prebiotic functions, positively influencing the gut microbiota and intestinal morphology, which, in turn, enhances gastrointestinal health and function [14,15]. The microorganisms used in the fermentation process synthesize an enzyme that breaks down phytate complexes—microbial phytase—which enhances the bioavailability not only of phosphorus and calcium [16,17] but also of other divalent minerals, such as copper, manganese, zinc, and iron [13]. Fermentation also produces bioactive compounds that help alleviate oxidative stress, a factor known to influence the development of bone and joint diseases [18,19].

Numerous studies have confirmed the beneficial effects of fermented products, highlighting their nutritional value—such as providing sulfur amino acids and more digestible protein and reducing anti-nutritional factors—as well as their health-promoting properties, including them being a source of antioxidant compounds, beneficial microorganisms, and digestive enzymes. Most of this research, however, has concentrated on the positive impacts of fermented products on the growth performance of pigs [12,20] and their antioxidant and immunomodulatory effects [14,15]. In contrast, relatively little attention has been paid to their effects on bone health, with few studies addressing how fermented feed additives influence the mechanical properties and mineralization of bones in weaned piglets [21,22].

Given the numerous advantages of fermented products and the potential benefits of incorporating them into piglets’ diets, this study aims to test the hypothesis that adding FRSM to the diet of weaned piglets may positively impact the mechanical properties and mineralization of their bones. To investigate this hypothesis, we analyzed the effects of different levels of dried fermented post-extraction rapeseed meal in piglet feed mixtures on the mineral absorption, bone osteometry, mineralization, and mechanical properties of piglet femora.

## 2. Materials and Methods

### 2.1. Animals and Experimental Design

This study was conducted on 18-day-old piglets (10 days before the planned weaning date), weighing an average of 5.29 kg [23]. The piglets (crossbreeds of Landrace and Yorkshire [LY] hybrids × Duroc and Pietrain [DP] hybrids) were born to 30 sows (at their second and third lactation). To ensure consistent experimental conditions, immediately after birth, the piglets were tagged and evenly distributed so that each sow had 14 piglets (7 gilts and 7 barrows). Subsequently, the sows and their litters were randomly divided into six groups, each consisting of piglets from five sows. The experimental factor was the different levels of dried FRSM introduced to replace post-extraction soybean meal (Table 1). The piglets in the FR-8 group received a feed mixture with 8% FRSM, the FR-12 group received 12%, the FR-15 group received 15%, and the FR-25 group received 25% (on a dry matter basis). Additionally, two control groups were established: the positive control (PC) group, with these piglets receiving a standard pre-starter mix supplemented with 2500 ppm zinc oxide, and the negative control (NC) group, with these piglets receiving a standard pre-starter mix without zinc oxide. All the diets were formulated to be iso-protein and iso-energetic. The process of rapeseed meal fermentation was previously described [23], and a detailed analysis of the nutritional value, mineral profile, and amino acid profile of FRSM is presented in [13]. On day 28 of life, the piglets were weaned from the sows. From each group, 50 piglets (with 10 selected from each litter, balanced for body weight and sex, and with 25 gilts and 25 barrows per group) were moved into a single pen for each treatment replicate in the weaner unit. FRSM inclusion did not negatively affect the growth rate (average daily gain) during either the pre-weaning or post-weaning periods, as previously reported in [23], where complete performance data are presented.

### 2.2. Fecal Collection and Digestibility Study

The digestibility analysis was performed when the piglets were 34 days old, utilizing the acid-insoluble ash (AIA) method [25]. Six piglets were randomly selected from each group, with one piglet chosen from each of the five replicate litters and an additional piglet selected randomly to ensure equal representation of gilts and barrows (three gilts and three barrows). The selected piglets were individually housed. The feed was supplemented with silicon dioxide (SiO_2_) as an inert marker at a concentration of 2 g/kg before pelleting [26].

Fecal samples were collected from each piglet in the morning on five consecutive days, seven days prior to slaughter, to assess the digestibility of the basic mineral components. The collected fecal samples were stored at 4 °C, and daily samples were weighed and dried to determine the drying coefficient. Concurrently, feed samples were also collected. The concentration of the SiO_2_ marker in both the feed and feces was determined using a gravimetric technique. The apparent total tract digestibility (ATTD) of the mineral components was calculated using the following formula:

ATTD = (1 − (N_f_∙M_d_)/(N_d_∙M_f_))∙100%, (1)

where N_d_ is the concentration of the nutrient in the diet, N_i_ is the concentration of the nutrient in the feces, M_d_ is the concentration of the marker in the diet, and M_f_ is the concentration of the marker in the feces, with all values expressed in g/kg dry matter (DM) [27].

### 2.3. Blood Sample Collection

On day 41 post-birth, prior to the first feeding, the piglets involved in the digestibility study were transported to a commercial slaughter facility. At this facility, the piglets were weighed and euthanized via electrical stunning, followed by exsanguination. Blood samples were collected into heparinized tubes and centrifuged at 4 °C at 1400× *g* for 10 min to obtain plasma. The plasma samples were then stored at −20 °C for subsequent analysis. The concentrations of calcium, zinc, copper, and iron in the blood plasma were measured using atomic absorption spectrometry, while the phosphorus content was determined colorimetrically.

### 2.4. Bone Collection and Mechanical Testing

Immediately after euthanasia, both femora were carefully dissected, cleaned of any adherent tissues, wrapped in gauze soaked in 0.9% saline, and placed separately into zip-lock bags before being frozen at −20 °C. For subsequent analyses, the right femora were used for the densitometry tests, mid-diaphysis geometry measurements, and mineral content assays, while the left femora were weighed, measured for their length, and subjected to a three-point bending testing.

The whole bone mineral content (BMC) and bone mineral density (BMD) were assessed using dual-energy X-ray absorptiometry (DXA) on a Lunar iDXA densitometer (GE, Madison, WI, USA), which was calibrated before each measurement using bone phantoms within the BMD range of 0.6–1.4 g/cm^2^. Following densitometry, the bones were sectioned using a diamond bandsaw (MBS 240/E, Proxxon GmbH, Foehren, Germany). The external and internal diameters of the mid-diaphysis cross-section were measured in both the transverse and anteroposterior planes using a digital caliper (1108–150, Insize Europe, Zamudio, Spain). Based on these measurements, the mid-diaphysis mean relative wall thickness (MRWT), cortical index (CI), cross-sectional area (CSA), and cross-sectional moment of inertia (Ix) were calculated [28]. Following these measurements, the bone mid-diaphysis of the femur was washed, defatted, and dried overnight at 105 °C. 

Before mechanical testing, the bones were allowed to thaw overnight at 4 °C. Following thawing, the bones were measured for their length using a ruler with an accuracy of 1 mm and weighed on a digital precision scale. The Seedor index, which is the ratio of bone weight to bone length, was then calculated. A quasi-static three-point bending test was performed using a universal testing machine (ZwickRoell 005, ZwickRoell GmbH & Co., Ulm, Germany) under the following conditions: a support span of 36 mm (equivalent to 40% of the average bone length, rounded to the nearest millimeter) and a loading rate of 10 mm/min. The load–deformation curves generated during testing were analyzed using Origin software (ver. 2022, OriginLab, Northampton, MA, USA). This analysis enabled the determination of key bone structural properties, including yield load, elastic work, fracture load, work to fracture, and stiffness. These structural traits, along with the geometrical parameters of the bone mid-diaphysis, were subsequently used to calculate the material properties of the bone, including Young’s modulus, elastic and fracture strain, and elastic and fracture stress [29].

### 2.5. Determination of Macro- and Microelement Content in Feces and Bones

The percentage of ash as well as the content of macro- and microelements, including calcium, phosphorus, copper, magnesium, iron, and zinc, in the feces and bone samples were determined using the standard procedures outlined by the AOAC [30].

### 2.6. Statistical Analysis

One-way ANOVA was performed to analyze the data, with treatment as the fixed effect and pig as the experimental unit (n = 6). Post hoc comparisons were conducted using Tukey’s HSD test. Prior to the analysis, the ANOVA assumptions, including the normality of the data distribution (verified by the Shapiro–Wilk test) and equality of variances (verified by Levene’s test), were confirmed. For the FRSM treatments (NC, FR-8, FR-12, FR-15, and FR-25), orthogonal polynomial contrasts were employed to assess the linear and quadratic effects of varying the FRSM levels on specific response variables. A *p*-value of less than 0.05 was considered statistically significant for all tests. The data analysis was carried out using Statistica software (v. 13.3, TIBCO Software Inc., Palo Alto, CA, USA).

## 3. Results

The inclusion of dried FRSM in the diet of the piglets positively affected the ATTD coefficients for the minerals (Table 2). The digestibility of phosphorus in the FR-25 group significantly surpassed that in the PC, NC, and FR-8 groups (*p* < 0.001), showing a linear trend with increasing levels of FRSM (*p* < 0.05). For all the other analyzed minerals, the ATTD coefficients for the FR-8 to FR-25 groups were significantly improved compared to both control groups, the PC and NC (*p* < 0.001), showing significant quadratic effects for calcium and magnesium (*p* < 0.05 for both) and both linear (*p* < 0.05 for iron and *p* < 0.001 for copper and zinc) and quadratic effects (*p* < 0.001 for all) for copper, iron, and zinc.

The inclusion of dried FRSM in the diet of the piglets did not significantly affect the geometric properties of their femora, as shown in Table 3. There were no significant differences observed in bone weight, bone length, the Seedor index, MRWT, CI, CSA, Ix, BMD, or BMC among the different treatment groups (*p* > 0.05). The statistical analysis confirmed no significant linear or quadratic trends across the different FRSM levels for any of the measured bone properties (*p* > 0.05). 

The mechanical properties of the femora in the piglets are presented in Table 4. The inclusion of dried FRSM in their diet had a significant impact on the structural parameters of their femora, which can generally be characterized as an improvement in the groups supplemented with intermediate doses of FRSM, namely the FR-12 and FR-15 groups, when compared to both control groups and the FR-25 group supplemented with the highest dose of FRSM, showing quadratic trends for FRSM inclusion for all traits. Specifically, the yield load was significantly higher in the FR-12 group compared to that in the FRSM-deprived NC group and the FR-25 group with the highest FRSM supplementation (*p* < 0.01). Elastic work was significantly higher in the FR-8 to FR-15 groups than in the NC and FR-25 groups (*p* < 0.001). The highest fracture loads were observed in the FR-12 and FR-15 groups (*p* < 0.001), which were both higher than in that in the FR-25 group, with the fracture load in the FR-12 group also higher than that in both control groups and the FR-8 group. However, work to fracture in the FR-12 to FR-25 groups was higher than in the other groups (*p* < 0.001), and this trait also showed the linear effect (*p* < 0.01) of the FRSM dose. The changes in stiffness were also significant (*p* < 0.01), with higher values observed for the intermediate doses of FRSM. However, when analyzing the material properties of the femora, a significant effect of the dietary treatment was only observed for fracture stress (*p* < 0.01), with the FR-12 group showing a significantly higher value compared to the PC and FR-8 and FR-25 groups, resulting in a weak but significant quadratic trend (*p* < 0.05). There were no significant differences observed in the other material properties (Young’s modulus, elastic strain, yield stress, or fracture strain) among the treatment groups (*p* > 0.05).

The contents of crude ash and mineral elements in the femora of the piglets, expressed on a dry matter basis, are presented in Table 5. The crude ash percentage and phosphorus, calcium, and iron contents in the FR-15 group were significantly greater than in the NC, FR-8, and FR-12 groups (*p* < 0.05 and *p* < 0.001, respectively), showing linear (*p* < 0.05 for calcium and iron) and quadratic effects (*p* < 0.001 and *p* < 0.01, respectively) of FRSM inclusion. Similarly, the highest contents of magnesium and zinc were observed in the FR-15 group (*p* < 0.001), and the lowest were seen in both the PC and NC groups and the FR-25 group, with significant linear and quadratic effects (*p* < 0.001 for both) of the FRSM dose. The femur copper content was higher in the FR-15 group than in the FR-12 and FR-25 groups (*p* < 0.01).

The inclusion of dried FRSM significantly affected the levels of minerals in the blood plasma of the piglets (Table 6), except for magnesium, whose levels showed no significant differences among the treatment groups (*p* > 0.05). The phosphorus levels were significantly higher in the FR-12 and FR-25 groups compared to the PC and NC groups (*p* < 0.001), showing both linear (*p* < 0.01) and quadratic (*p* < 0.001) effects of FRSM inclusion. Th calcium levels were significantly higher in the FR-8 group compared to those in the FR-15 group (*p* < 0.01). The copper levels were significantly higher in the FR-25 group compared to the PC and NC groups (*p* < 0.001). The iron levels in all of the FRSM-containing groups were similar and significantly higher compared to those in the PC and NC groups (*p* < 0.001). The zinc levels were significantly lower in the NC and FR-8 groups compared to all the other groups (*p* < 0.001), showing a significant linear effect (*p* < 0.01) of FRSM inclusion.

## 4. Discussion

Rapeseed meal, despite its favorable amino acid profile, high protein content, and rich mineral composition, faces limitations in its utilization due to its content of anti-nutritional factors [31]. However, the fact that rapeseed meal is a non-GMO ingredient has made it a focus of interest in terms of exploiting its nutritional potential. This study specifically explored the use of FRSM in the diet of weaned piglets, a critical period during which the digestibility and absorption of minerals are vital due to their rapid growth and underdeveloped digestive systems [32].

Weaned piglets are particularly susceptible to stress, dietary changes, and infections, all of which can damage the intestinal epithelium—a key site for nutrient absorption [1,2,33,34]. Fermented products contribute to lowering the pH of the gastrointestinal tract, which inhibits the growth of pathogenic microorganisms, protects the intestinal lining from damage, and helps maintain its functionality [35]. Moreover, fermentation is recognized as one of the most effective methods for reducing anti-nutritional factors; it can decrease crude fiber, glucosinolates, and phytic acid by 25.5%, 43.1%, and 42.4%, respectively [36]. This reduction is promising for enhancing the bioavailability of nutrients in feed mixtures. Additionally, fermented components increase the activity of digestive enzymes such as lipase, amylase, and protease, thereby contributing to improved gut health [36].

A healthy gut microbiota enhances the availability, absorption, and digestion of nutrients and minerals [37,38]. Research shows that FRSM positively influences the gut microbiota by modulating the microflora, reducing harmful strains such as *E. coli*, *Staphylococcus* spp., *Enterococcus faecalis*, and *Listeria monocytogenes* while increasing beneficial probiotic strains like *Lactobacillus* spp. [39]. Other studies have underscored the positive impact of fermented products on immune system function, which leads to a stronger intestinal barrier and improved mineral bioavailability [40].

In this study, it was observed that FRSM increased the digestibility of essential minerals, including phosphorus (25% FRSM), calcium, magnesium, copper, zinc (12–25% FRSM), and iron (15–25% FRSM). Similar findings were reported by Konkol et al. [41], who noted a reduced phosphorus content in the feces of laying hens fed FRSM, and by Shi et al. [42], who observed the same trend in growing pigs. Increased mineral digestibility may be linked to a reduction in anti-nutritional factors in the feed mixtures [36]. Phytic acid, a significant anti-nutritional factor in monogastric animals, has strong chelating properties, forming insoluble complexes with minerals and thereby limiting their availability [5]. Fermented components help reduce phytic acid levels The observed increase in mineral digestibility may also have resulted from the acidification of the gastrointestinal contents by lactic acid bacteria, which enhances the activity of endogenous phytases in plant material and creates optimal pH conditions for endogenous phytase activity [43].

The prebiotic and probiotic effects of fermented products, achieved through the modulation of the gut microbiota, may also contribute to improved bone characteristics [44]. In this study, the piglets fed a diet containing 15% FRSM exhibited increased mineral content in their femora, including higher levels of phosphorus, calcium, magnesium, copper, zinc, and iron. These findings are consistent with earlier studies by Czech et al. [45] which demonstrated that piglets receiving fermented rapeseed and soybean meal had a significantly higher mineral content in both their blood plasma and metacarpal bones.

The enhanced mineral levels in their bones can be attributed to the reduced gastrointestinal pH resulting from the presence of *Lactobacillus*, which improve mineral bioavailability [38]. Furthermore, the lactic acid produced during fermentation can positively influence the intestinal morphology. Specifically, FRSM has been shown to increase the height of the intestinal villi and the depth of their crypts, which are important factors in nutrient absorption [46]. Previous studies have confirmed that a higher villus-height-to-crypt-depth ratio is associated with improved absorption and digestion in piglets [12,47]. Additionally, the short-chain fatty acids (SCFAs) produced during fermentation stimulate the proliferation of the intestinal cells, thereby increasing the absorptive surface area and further enhancing nutrient absorption [37]. 

Calcium is a crucial substrate for bone formation and plays a vital role in bone metabolism [48]. The appropriate calcium-to-phosphorus standardized total tract digestible (STTD) ratio is also essential for optimal growth, with the recommended ratios in pigs being 1.39:1 or 1.25:1 to achieve the maximum growth [49,50]. Calcium and phosphorus work synergistically in promoting proper bone growth and development [8,51], with extracellular inorganic phosphorus and ionic calcium concentrations being key determinants of bone mineralization [52].

In this study, an increase in femoral calcium content was particularly notable in the group receiving 15% FRSM, while the plasma calcium levels only increased in the 8% FRSM group, with the other groups showing reduced levels or comparable levels to the control group. This discrepancy may be partially explained by the role of SCFAs in enhancing calcium bioavailability by increasing the expression of intracellular calcium transporters. Enhanced calcium absorption can lead to reduced secretion of parathyroid hormone, which, in turn, lowers blood calcium levels and increases calcium deposition in the bones [37].

Copper and zinc also play crucial roles in collagen fiber formation and the enzymatic reactions necessary for healthy bone tissue modeling [53]. However, these two elements can act antagonistically [54], which may have accounted for the observed increase in copper levels and decrease in zinc levels in the piglets’ plasma, especially in the 8% FRSM group compared to the control group.

The mechanical properties of bones are influenced not only by their mineral content but also by their organic components, which include collagen and other organic proteins that make up approximately 30% of bone mass. While mineral components, primarily calcium and phosphorus deposited as hydroxyapatite, provide bones with hardness and mechanical strength, collagen fibers impart flexibility and resilience. The organic phase, which serves as a scaffold for the mineral phase, imparts elasticity and allows bones to absorb impacts without breaking easily [22,55]. The interaction and balance between these phases and the quality of these components are essential for maintaining bone integrity, as the rigidity of the mineral phase, combined with the elasticity of the organic phase, ensures that bones can withstand various mechanical stresses [56].

The absence of significant changes in bone mid-shaft mineralization (BMD/BMC), bone weights, or cross-sectional geometry suggests that the noted improvements in the bones’ mechanical properties were likely related to modifications in their organic phase structure, particularly the organization of collagen fibers. An increase in femoral stiffness was observed in the piglets fed FRSM, although this did not correlate with Young’s modulus, a material property that reflects bone stiffness as a structural trait. While no significant changes in Young’s modulus were detected, the observed increase in yield load might indicate stimulated collagen synthesis, potentially influenced by FRSM components like copper and zinc. It is well documented that copper and zinc play roles in collagen synthesis [57], and the increased zinc content observed in this study likely contributed to the production of new, immature collagen fibers [54,58]. Collagen content correlates with the yield point, which marks the transition from elastic to plastic deformation of bone under a load [57]. The observed increase in the yield point deformation in the piglets receiving the FRSM diet suggests enhanced collagen synthesis. Additionally, fermented products may support the synthesis of amino acids such as glutamine, proline, and hydroxyproline, which make up 57% of the proteins in collagen [59], further promoting collagen synthesis and potentially affecting bone structure and function. However, a primary limitation of the present study is the lack of a mineral composition analysis of the soybean meal and the FRSM used in these diets. Without precise mineral content data, it is challenging to fully ascertain the specific contributions of these feed ingredients to the mineral intake and bioavailability in the piglets.

On the other hand, the changes in fracture load, work to fracture, and fracture stress—which correspond to the maximum stress a bone can withstand before fracturing—indicate potential alterations in the structural organization and size of the bone hydroxyapatites. These changes may influence the internal stresses in the mineral phase, which are critical for load sharing in bones and their fracture strength [60]. Interestingly, changes in hydroxyapatite size have previously been observed in the bones of weaners maternally supplemented with FRSM [21].

Such changes could be due to the substitution of calcium ions in hydroxyapatites with other cations present in the bone mineral phase, a process known to impact bones’ mechanical properties [61]. For example, magnesium and zinc substitutions have been shown to alter the crystallographic parameters of hydroxyapatite crystallites [62], leading to a reduced crystal size, which is beneficial for bone mechanical properties, likely by preventing crack propagation and increasing bone stiffness [61,63,64]. In this study, the femora of pigs from the FR-8, FR-12, and FR-15 groups, which had increased magnesium and zinc levels, exhibited greater stiffness. Future studies should investigate these aspects further using not only histological and immunohistochemical analyses but also physical techniques such as FTIR, AFM, XRD, SAXS, or DSC analysis, which could provide additional insights into the structural organization of both the organic and mineral phases of bone [21,55,60,65,66,67].

## 5. Conclusions

The inclusion of dried FRSM in the diet of weaned piglets significantly enhances their mineral absorption and bone health. Intermediate levels of FRSM (12–15%) improve the digestibility of essential minerals including phosphorus, calcium, magnesium, copper, zinc, and iron and support the structural integrity of bones by increasing their mineral content and likely promoting collagen synthesis. Additionally, FRSM helps to reduce anti-nutritional factors, thereby creating a healthier intestinal environment that is conducive to optimal nutrient absorption. As a non-GMO feed component, FRSM presents a valuable alternative for enhancing animal growth and overall health. Future research should aim to optimize the FRSM levels to maximize these benefits and further explore its potential in animal nutrition.

## Figures and Tables

**Table 1:** Basal components and nutritional values of the diets [23,24].

Item/Diet ^1^	NC	PC	FR-8	FR-12	FR-15	FR-25
Fermented rapeseed meal	0	0	8	12	15	25
Barley	35	35	35	35	35	35
Wheat	33.69	33.24	29.69	26.97	26.08	19.36
Soybean meal	13	13.2	3.9	4.0	2.4	0
Potato protein	2.5	2.5	2.5	2.5	2.5	2.5
Fishmeal	3	3	3	3	3	1.4
Whey powder	3	3	4.25	4.25	4.25	4.25
Whole milk powder	5	5	5	5	5	5
Vegetable oil	0.7	0.7	4.25	2.87	2.93	3.7
L-Lys (98.5%)	0.74	0.73	0.7	0.76	0.76	0.3
DL-Met (99%)	0.22	0.22	0.21	0.2	0.2	0.2
L-Thr (93.5%)	0.3	0.3	0.3	0.29	0.29	0.3
L-Trp (98%)	0.12	0.12	0.15	0.15	0.15	0.16
Monocalcium phosphate	0.91	0.92	1.02	0.94	0.38	0.78
Sodium chloride	0.25	0.25	0.31	0.35	0.34	0.33
Calcium formate	0.4	0.4	0.7	0.7	0.7	0.7
Sodium bicarbonate	0.15	0.15	0.0	0.0	0.0	0.0
Iron fumarate 31%	0.25	0.25	0.25	0.25	0.25	0.25
Piger dry aroma	0.2	0.2	0.2	0.2	0.2	0.2
EP premix ^2^	0.5	0.5	0.5	0.5	0.5	0.5
Sucram	0.07	0.07	0.07	0.07	0.07	0.07
Medicinal ZnO	0.0	0.25	0.0	0.0	0.0	0.0
*Calculated values*						
ME (MJ/kg)	13.9	13.4	14.2	14.1	13.5	13.9
*Analyzed values* ^A^						
Dry matter (g/kg)	87.96	88.33	87.87	87.99	88.42	88.07
Crude ash (g/kg)	5.69	6.01	5.72	5.88	6.08	5.87
Crude protein (g/kg)	19.13	19.08	18.66	18.62	18.49	18.64
Crude fat (g/kg)	5.64	5.66	6.89	7.01	6.97	7.37
Crude fiber (g/kg)	4.01	3.99	3.38	3.62	3.79	4.32
Calcium (g/kg)	6.44	6.37	7.05	7.14	7.38	7.57
Phosphorus (g/kg)	6.25	6.23	6.58	6.57	6.59	6.58
Magnesium (g/kg)	1.20	1.23	1.28	1.29	1.32	1.40
Copper (mg/kg)	125.3	125.9	130.1	132.2	131.6	133.4
Zinc (mg/kg)	21.0	156.3	22.6	23.4	23.6	24.8
Iron (mg/kg)	180.4	181.3	183.6	184.3	184.9	185.3

^1^ NC—negative control; PC—positive control (0.25% ZnO = PC). ^2^ Nuklospray^®^ E50 provided in the pre-starter diets for the NC and PC; 0.25% Arbocel^®^ and 0.10% Globigen^®^ Jump Start provided in the pre-starter for the NC; and 0.5% microbial protein, 0.03% Xylanase, 0.03% Ronozyme^®^ VP, 0.1% Mycofix^®^ Plus, 0.5% Tetracid^®^ Dry acid, 0.1% ProPen H, 0.2% Acidomatrix™, and 12000 IU beta-xylanase provided in the diets for the NC and PC. ^A^ Based on data analyzed and reported previously [23].

**Table 2:** Apparent total tract digestibility (ATTD, %) coefficients of macro- and microelements.

Treatment ^1^	Phosphorus	Calcium	Magnesium	Copper	Iron	Zinc
PC ^2^	46.05 ^c^	36.74 ^b^	59.91 ^b^	40.96 ^b^	17.70 ^c^	30.51 ^b^
NC ^3^	46.12 ^bc^	36.00 ^b^	58.07 ^b^	28.17 ^c^	12.61 ^d^	18.83 ^c^
FR-8	45.45 ^bc^	38.29 ^ab^	61.75 ^ab^	41.75 ^b^	21.62 ^b^	32.13 ^b^
FR-12	50.91 ^ab^	40.57 ^a^	64.40 ^a^	45.56 ^a^	24.10 ^ab^	35.33 ^a^
FR-15	50.56 ^ab^	41.57 ^a^	64.81 ^a^	44.06 ^a^	26.09 ^a^	36.29 ^a^
FR-25	52.55 ^a^	40.96 ^a^	64.77 ^a^	45.06 ^a^	28.72 ^a^	36.97 ^a^
SEM ^4^	0.637	0.464	0.548	1.053	0.938	1.071
*p*-value						
TRT ^5^	<0.001	<0.001	<0.001	<0.001	<0.001	<0.001
FR ^6^	<0.001	<0.001	<0.001	<0.001	<0.001	<0.001
Linear ^7^	0.020	0.633	0.328	<0.001	0.048	<0.001
Quadratic ^8^	0.987	0.013	0.001	<0.001	<0.001	<0.001

^a–d^ Means with the same superscript are statistically the same across all 6 treatments (*p* > 0.05) based on Tukey’s post hoc test. ^1^ There were a total of 5 dietary treatments. Diets NC–FR-25 were ZnO-free diets with 0 (NC), 8%, 12%, 15%, and 25% FRSM, respectively. The PC (positive control) diet was formulated on the basis of a basic mixture (NC) with 2.5% ZnO. ^2^ PC—positive control; ^3^ NC—negative control; ^4^ SEM—standard error of the means. ^5^ *p*-value for overall effect of dietary treatment (diets PC-FR-25). ^6^ *p*-value for effect of FRSM (diets NC-FR-25). ^7,8^ Orthogonal polynomial (linear and quadratic) contrasts were performed to test the effect of FRSM.

**Table 3:** Osteometric, geometric, and densitometric characteristics of the femora obtained from piglets at the age of 41 d.

Treatment ^1^	Bone Weight, g	Bone Length, mm	Seedor Index, g/cm	MRWT, --	CI, %	CSA, mm^2^	Ix, Mm^4^	BMD, g/cm^2^	BMC, g
PC ^2^	27.6	87.8	3.19	0.937	47.3	73.2	500	0.421	5.20
NC ^3^	23.1	84.8	2.71	1.023	48.1	62.9	396	0.398	5.67
FR-8	25.9	87.5	3.00	0.898	46.3	74.6	563	0.408	5.77
FR-12	26.9	88.0	3.11	1.122	51.6	73.1	458	0.422	5.92
FR-15	27.9	87.7	3.20	0.947	48.1	78.3	638	0.377	5.53
FR-25	24.6	85.8	2.88	0.897	46.4	70.3	532	0.369	5.42
SEM ^4^	2.31	2.84	0.311	0.1056	2.51	5.42	57.2	0.023	0.502
*p*-value									
TRT ^5^	0.664	0.956	0.854	0.679	0.701	0.485	0.086	0.469	0.934
FR ^6^	0.461	0.897	0.702	0.529	0.550	0.376	0.072	0.206	0.970
Linear ^7^	0.533	0.826	0.596	0.493	0.743	0.353	0.111	0.140	0.734
Quadratic ^8^	0.096	0.333	0.202	0.662	0.440	0.101	0.145	0.231	0.650

^1^ There were a total of 5 dietary treatments. Diets NC–FR-25 were ZnO-free diets with 0 (NC), 8%, 12%, 15%, and 25% FRSM, respectively. A PC (positive control) diet was formulated on the basis of a basic mixture (NC) with 2.5% ZnO. MRWT—mid-diaphysis mean relative wall thickness; CI—cortical index (CI); CSA—cross-sectional area; Ix—cross-sectional moment of inertia; BMD—bone mineral density; BMC—bone mineral content. ^2^ PC—positive control; ^3^ NC—negative control; ^4^ SEM—standard error of the means. ^5^ *p*-value for overall effect of dietary treatment (diets PC-FR-25). ^6^ *p*-value for effect of FRSM (diets NC-FR-25). ^7,8^ Orthogonal polynomial (linear and quadratic) contrasts were performed to test the effect of FRSM.

**Table 4:** Mechanical characteristics of femora obtained from piglets at the age of 42 d.

Treatment ^1^	Structural Properties					Material Properties				
	Yield Load, N	Elastic Work, mJ	Fracture Load, N	Work to Fracture, mJ	Stiffness, N/mm	Young’s Modulus, MPa	Elastic Strain, %	Yield Stress, MPa	Fracture Strain, %	Fracture Stress, MPa
PC ^2^	496 ^ab^	599 ^ab^	622 ^ab^	1198 ^a^	240 ^a^	485	9.71	41.3	15.6	51.2 ^a^
NC ^3^	480 ^a^	448 ^a^	634 ^ab^	1316 ^a^	269 ^ab^	684	7.45	50.0	12.9	66.0 ^ab^
FR-8	568 ^ab^	671 ^b^	710 ^ab^	1457 ^a^	392 ^c^	557	8.02	43.5	16.5	53.7 ^a^
FR-12	699 ^b^	696 ^b^	950 ^c^	2331 ^b^	382 ^bc^	798	8.21	62.1	15.3	83.4 ^b^
FR-15	623 ^ab^	737 ^b^	832 ^bc^	2086 ^b^	317 ^abc^	530	9.07	44.2	16.8	60.2 ^ab^
FR-25	449 ^a^	464 ^a^	591 ^a^	2005 ^b^	253 ^ab^	566	7.91	41.8	16.7	51.0 ^a^
SEM ^4^	47.8	57.0	51.0	115.1	30.0	107.4	0.812	5.92	1.17	5.56
*p*-value										
TRT ^5^	0.007	0.003	<0.001	<0.001	0.003	0.346	0.403	0.143	0.189	0.002
FR ^6^	0.009	0.004	<0.001	<0.001	0.015	0.370	0.633	0.167	0.178	0.005
Linear ^7^	0.725	0.871	0.785	<0.001	0.366	0.455	0.535	0.398	0.0.54	0.141
Quadratic ^8^	<0.001	<0.001	<0.001	0.003	0.002	0.867	0.268	0.384	0.266	0.046

^a–c^ Means with the same superscript are statistically the same across all 6 treatments (*p* > 0.05) based on Tukey’s post hoc test. ^1^ There were a total of 5 dietary treatments. Diets NC–FR-25 were ZnO-free diets with 0 (NC), 8%, 12%, 15%, and 25% FRSM, respectively. A PC (positive control) diet was formulated on the basis of a basic mixture (NC) with 2.5% ZnO. ^2^ PC—positive control; ^3^ NC—negative control; ^4^ SEM—standard error of the means. ^5^ *p*-value for overall effect of dietary treatment (diets PC-FR-25). ^6^ *p*-value for effect of FRSM (diets NC-FR-25). ^7,8^ Orthogonal polynomial (linear and quadratic) contrasts were performed to test the effect of FRSM.

**Table 5:** Bone mineral contents in femora obtained from piglets at the age of 41 d (dry matter basis).

Treatment ^1^	Crude ash, %	Phosphorus, g/kg	Calcium, g/kg	Magnesium, g/kg	Copper, mg/kg	Iron, mg/kg	Zinc, mg/kg
PC ^2^	42.85 ^ab^	74.17 ^ab^	169.7 ^b^	6.37 ^c^	0.335 ^ab^	16.43 ^b^	115.2 ^c^
NC ^3^	41.23 ^b^	70.42 ^c^	164.5 ^c^	6.06 ^d^	0.343 ^ab^	16.31 ^b^	113.9 ^c^
FR-8	41.38 ^b^	72.97 ^bc^	166.6 ^bc^	6.38 ^c^	0.345 ^ab^	16.59 ^ab^	115.8 ^c^
FR-12	42.58 ^b^	72.53 ^bc^	169.1 ^bc^	6.59 ^b^	0.313 ^b^	16.60 ^ab^	118.1 ^b^
FR-15	43.20 ^a^	76.96 ^a^	175.8 ^a^	6.85 ^a^	0.357 ^a^	17.13 ^a^	122.9 ^a^
FR-25	42.09 ^ab^	74.21 ^ab^	169.1 ^bc^	6.37 ^c^	0.324 ^b^	16.42 ^b^	115.2 ^c^
SEM ^4^	0.218	0.425	0.673	0.044	0.004	0.067	0.540
*p*-value							
TRT ^5^	0.033	<0.001	<0.001	<0.001	0.004	<0.001	<0.001
FR ^6^	0.029	<0.001	<0.001	<0.001	0.002	0.003	<0.001
Linear ^7^	0.836	0.070	0.023	<0.001	0.081	0.026	<0.001
Quadratic ^8^	0.066	0.008	<0.001	<0.001	0.671	0.003	<0.001

^a–d^ Means with the same superscript are statistically the same across all 6 treatments (*p* > 0.05) based on Tukey’s post hoc test. ^1^ There were a total of 5 dietary treatments. Diets NC–FR-25 were ZnO-free diets with 0 (NC), 8%, 12%, 15%, and 25% FRSM, respectively. A PC (positive control) diet was formulated on the basis of a basic mixture (NC) with 2.5% ZnO. ^2^ PC—positive control; ^3^ NC—negative control; ^4^ SEM—standard error of the means. ^5^ *p*-value for overall effect of dietary treatment (diets PC-FR-25). ^6^ *p*-value for effect of FRSM (diets NC-FR-25). ^7,8^ Orthogonal polynomial (linear and quadratic) contrasts were performed to test the effect of FRSM.

**Table 6:** Blood plasma macro- and microelement concentration in piglets at the age of 42 d.

Treatment ^1^	Phosphorus, mmol/L	Calcium, mmol/L	Magnesium, mmol/L	Copper, μmol/L	Iron, μmol/L	Zinc, μmol/L
PC ^2^	1.72 ^c^	2.53 ^ab^	1.19	16.34 ^c^	20.29 ^b^	11.83 ^a^
NC ^3^	1.84 ^c^	2.64 ^ab^	1.26	18.44 ^bc^	22.28 ^b^	8.16 ^b^
FR-8	2.54 ^b^	2.83 ^a^	1.32	20.52 ^ab^	27.45 ^a^	9.56 ^b^
FR-12	2.82 ^a^	2.61 ^ab^	1.38	20.27 ^ab^	30.00 ^a^	11.61 ^a^
FR-15	2.53 ^b^	2.30 ^b^	1.40	19.49 ^ab^	28.92 ^a^	11.22 ^a^
FR-25	2.69 ^ab^	2.66 ^ab^	1.37	22.23 ^a^	31.55 ^a^	11.62 ^a^
SEM ^4^	0.075	0.038	0.024	0.432	0.811	0.285
*p*-value						
TRT ^5^	<0.001	0.001	0.085	<0.001	<0.001	<0.001
FR ^6^	<0.001	0.001	0.488	0.031	<0.001	0.010
Linear ^7^	0.002	0.530	0.775	0.812	0.684	0.002
Quadratic ^8^	<0.001	0.191	0.331	0.709	0.069	0.131

^a–c^ Means with the same superscript are statistically the same across all 6 treatments (*p* > 0.05) based on Tukey’s post hoc test. ^1^ There were a total of 5 dietary treatments. Diets NC–FR-25 were ZnO-free diets with 0 (NC), 8%, 12%, 15%, and 25% FRSM, respectively. A PC (positive control) diet was formulated on the basis of a basic mixture (NC) with 2.5% ZnO. ^2^ PC—positive control; ^3^ NC—negative control; ^4^ SEM—standard error of the means. ^5^ *p*-value for overall effect of dietary treatment (diets PC-FR-25). ^6^ *p*-value for effect of FRSM (diets NC-FR-25). ^7,8^ Orthogonal polynomial (linear and quadratic) contrasts were performed to test the effect of FRSM.

## Data Availability

The data presented in this study are available on request from the corresponding authors.
